# Genome-wide 5-Hydroxymethylcytosine Profiling Analysis Identifies MAP7D1 as A Novel Regulator of Lymph Node Metastasis in Breast Cancer

**DOI:** 10.1016/j.gpb.2019.05.005

**Published:** 2021-03-11

**Authors:** Shuang-Ling Wu, Xiaoyi Zhang, Mengqi Chang, Changcai Huang, Jun Qian, Qing Li, Fang Yuan, Lihong Sun, Xinmiao Yu, Xinmiao Cui, Jiayi Jiang, Mengyao Cui, Ye Liu, Huan-Wen Wu, Zhi-Yong Liang, Xiaoyue Wang, Yamei Niu, Wei-Min Tong, Feng Jin

**Affiliations:** 1 Department of Surgical Oncology and Breast Surgery, the First Affiliated Hospital of China Medical University, Shenyang 110000, China; 2 Department of Pathology, Institute of Basic Medical Sciences Chinese Academy of Medical Sciences, School of Basic Medicine Peking Union Medical College, Molecular Pathology Research Center, Chinese Academy of Medical Sciences, Beijing 100005, China; 3 State Key Laboratory of Medical Molecular Biology, Department of Biochemistry and Center for Bioinformatics, Institute of Basic Medical Sciences Chinese Academy of Medical Sciences, School of Basic Medicine Peking Union Medical College, Beijing 100005, China; 4 Beijing National Laboratory for Molecular Sciences (BNLMS), MOE Key Laboratory of Bioorganic Chemistry and Molecular Engineering, College of Chemistry, Peking University, Beijing 100871, China; 5 Center for Experimental Animal Research, Institute of Basic Medical Sciences Chinese Academy of Medical Sciences, School of Basic Medicine Peking Union Medical College, Beijing 100005, China; 6 Department of Pathology, Peking Union Medical College Hospital, Chinese Academy of Medical Sciences, Peking Union Medical College, Molecular Pathology Research Center, Chinese Academy of Medical Sciences, Beijing 100005, China

**Keywords:** 5-Hydroxymethylcytosine, Breast cancer, Lymph node metastasis, MAP7D1, Biomarker

## Abstract

Although DNA **5-hydroxymethylcytosine (5hmC)** is recognized as an important epigenetic mark in cancer, its precise role in **lymph node metastasis** remains elusive. In this study, we investigated how 5hmC associates with lymph node metastasis in **breast cancer**. Accompanying with high expression of TET1 and TET2 proteins, large numbers of genes in the metastasis-positive primary tumors exhibit higher 5hmC levels than those in the metastasis-negative primary tumors. In contrast, the TET protein expression and DNA 5hmC decrease significantly within the metastatic lesions in the lymph nodes compared to those in their matched primary tumors. Through genome-wide analysis of 8 sets of primary tumors, we identified 100 high-confidence metastasis-associated 5hmC signatures, and it is found that increased levels of DNA 5hmC and gene expression of **MAP7D1** associate with high risk of lymph node metastasis. Furthermore, we demonstrate that MAP7D1, regulated by TET1, promotes tumor growth and metastasis. In conclusion, the dynamic 5hmC profiles during lymph node metastasis suggest a link between DNA 5hmC and lymph node metastasis. Meanwhile, the role of MAP7D1 in breast cancer progression suggests that the metastasis-associated 5hmC signatures are potential **biomarkers** to predict the risk for lymph node metastasis, which may serve as diagnostic and therapeutic targets for metastatic breast cancer.

## Introduction

Breast cancer is the most common type of cancers in women worldwide [1]. Although current approaches such as surgery, chemotherapy, endocrine, and targeted therapies have improved overall survival rate, prognosis for patients with metastatic diseases remains poor. Among various metastatic sites, lymph node is the most common one. The malignant cells originally residing within lymph nodes can exit the node, and subsequently invade local blood vessels [2], [3]. Thus, lymph node metastasis may serve as a source of distant metastases that links lymphatic metastasis to hematogenous metastasis. Lymphatic metastasis is a sophisticated process that comprises multiple biological mechanisms, including cancer cell dissociation, dissemination, lymph angiogenesis, and establishment of a metastatic focus [4], [5]. Considerable progress has been made in clarifying the molecular mechanisms driving lymph node metastasis, including chemokine-mediated signaling transduction, vascular endothelial growth factor (VEGF)-activated lymph angiogenesis [6], tumor-draining lymph node (TDLN) formation [7], and tumor-induced immune modulation [8]. Despite these advances, a more detailed understanding of the intrinsic molecular mechanisms of lymph node metastasis is essential for improving clinical management of breast cancer. Furthermore, lymph node metastasis is an early event in cancer metastasis and partially indicates the aggressiveness of cancer. Thereby, discovery of its associated molecular signatures will be of immense value for developing potential biomarkers to predict the risk of metastasis.

It is generally believed that both genetic and epigenetic alterations represent causative factors of cancer metastasis. Recent studies have shown that the majority of genetic aberrations in lymph node metastasis have already preexisted in primary tumors, while gene mutations or variations that are only present in metastatic lesions are very few [9], [10]. Therefore, it appears that metastases tend to possess more frequent epigenetic alterations, and epigenetic regulation is critically involved in cancer metastasis [4].

Cumulative evidence supports the role of DNA 5-methylcytosine (5mC) modification in the progression of multiple types of cancers [11], including breast cancer metastasis [12]. Multiple DNA methylation signatures have been identified to be associated with breast cancer metastasis and prognosis. For example, a breast CpG island methylator phenotype (B-CIMP) has been identified from a systematic methylome analysis of breast cancer with diverse metastatic behavior [13]. Another cell-free circulating tumor DNA (ctDNA)-based methylation analysis has identified a subset of genes whose high methylation correlates with shorter survival, potentially suitable for predicting patient prognosis [14].

DNA 5-hydroxymethylcytosine (5hmC) is an oxidative product of 5mC, and functions as an independent epigenetic mark in various biological processes. Notably, a number of earlier studies have reported that DNA 5hmC levels exhibit significant reduction in multiple types of cancers [15]. Genome-wide analyses have identified a large number of differentially hydroxymethylated regions (DhMRs) between tumors and their adjacent normal tissues [16], [17]. Such gene-specific 5hmC changes may affect gene expression, possibly by interfering with DNA-protein interactions, thus causally linking epigenetics with disease. Therefore, identifying these differentially hydroxymethylated genes and their downstream regulatory networks would help to elucidate mechanistic details of cancer initiation and progression. Importantly, 5hmC signatures in tumors are also regarded as robust candidate biomarkers for both diagnosis and prognosis in various types of cancers [18].

Ten-eleven translocation (TET) enzymes are key players in catalyzing the conversion of 5mC to 5hmC and participate in the control of cellular differentiation and transformation. Previous studies have shown that TET enzymes regulate breast cancer growth and metastasis through multiple cancer-related factors and pathways, including HOXA9, miR-200 [19], and TNFα-p38-MAPK [20]. Moreover, during the progression from ductal carcinoma *in situ* (DCIS) to invasive ductal carcinoma (IDC) in breast cancer, the nuclear expression of TET1 and TET2 decreased significantly, accompanied by a reduction in global DNA 5hmC level [21]. However, among the IDCs of different grades, the 5hmC level and the expression of TET1 and TET3 were higher in high-grade IDCs than in low-grade IDCs, suggesting the different functions of 5hmC in the processes of tumorigenesis and cancer development [20].

Given that high-grade IDCs are often accompanied by severe lymph node metastasis or distant metastasis, we asked whether and how 5hmC-related epigenetic regulation contributes to lymph node metastasis in breast cancer. In this study, extensive gene-specific 5hmC changes in exons were found in lymph node metastasis of breast cancer. We identified a positive correlation between the 5hmC level of *MAP7D1* and the metastatic ability of primary tumors and further demonstrated its regulatory effect on breast cancer progression.

## Results

### Higher DNA 5hmC levels present in lymph node metastasis-positive primary breast cancer

**Figure 1:**
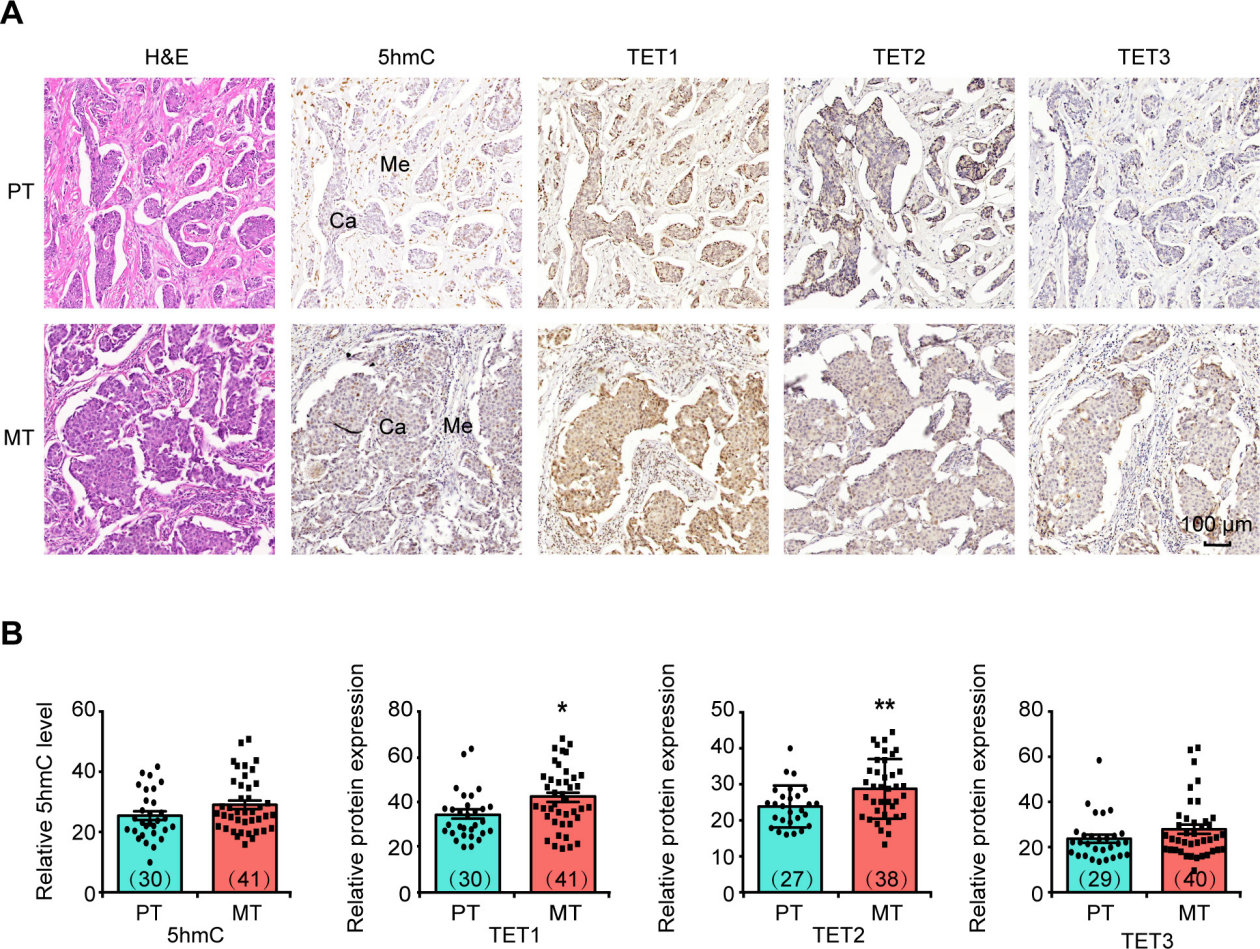
**Differences in 5hmC leveland TETprotein expressionlevelbetweenPT and MT lesions** **A.** Representative images of H&E staining and IHC staining to show the global DNA 5hmC level and expression of TET1, TET2, and TET3 in PT and MT tissues. Scale bar, 100 μm. **B.** Quantitative comparison of the levels of DNA 5hmC and TET1/2/3 protein expression between PT and MT tissues. The intensities of IHC staining in invasive ductal breast cancer cells but not in mesenchymal cells were analyzed for comparison. The number of samples used for immunostaining in each group was shown in the parenthesis. Data are represented as mean ± SEM. *P* values are calculated by using unpaired Student’s *t*-test (*, *P* < 0.05; **, *P* < 0.01). Ca represents invasive ductal breast cancer cells; Me represents mesenchymal cells. H&E staining, hematoxylin and eosin staining; IHC staining, immunohistochemical staining; PT, metastasis-negative primary tumor; MT, lymph node metastasis-positive primary tumor.

Lymph node metastasis is generally an early event in cancer metastasis. To investigate whether DNA 5hmC associates with this process, we performed immunostaining analysis of 5hmC and TET proteins using metastasis-negative primary tumor (PT) and lymph node metastasis-positive primary tumor (MT) samples (Figure 1A). Due to the existence of mesenchymal cells that exhibit different levels of DNA 5hmC and TET expression, we chose cancer cells only for quantitative analysis (Figure S1A and B). Compared with the PT group, the MT group only displayed a tendency of increase in its global 5hmC level and TET3 expression, but showed a significant increase in TET1 and TET2 expression (*P* < 0.05) (Figure 1B).

**Figure 2:**
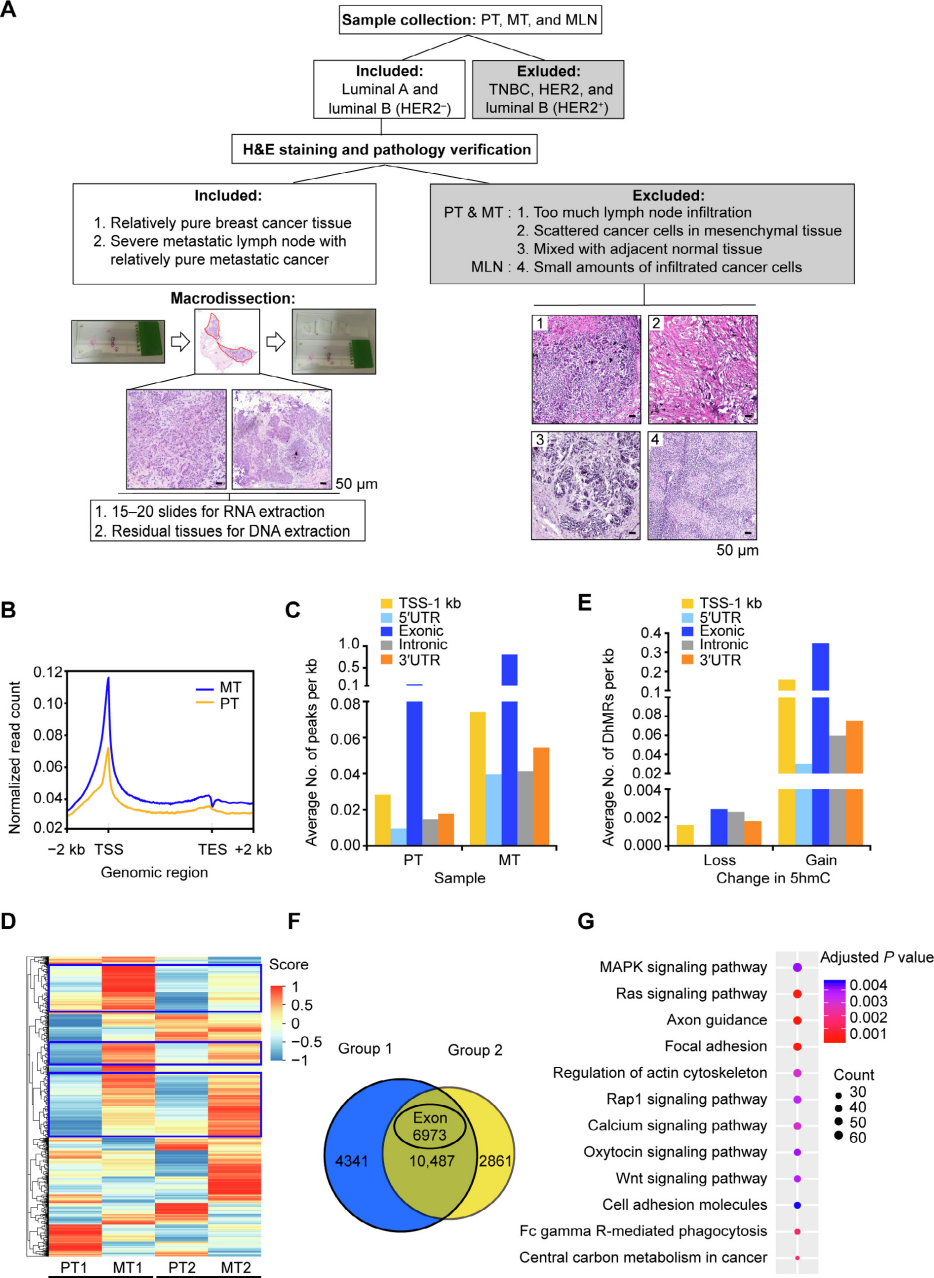
**Regional DNA 5hmC gainin MTlesionsduring breast cancer lymph node metastasis** **A.** Schematic representation of sample selection and macrodissection for all the samples used in this study. Luminal A and luminal B (HER2^−^) invasive ductal breast cancer tissues were selected for H&E staining and morphology analysis in order to distinguish tumor cells from mesenchymal cells and infiltrated immune cells. The red-circled area indicates the tumor cells collected for downstream analysis. The four types of non-tumor tissues excluded from this study were recognized based on the results of H&E staining as shown in images 1–4. Scale bar, 50 μm. **B.** Distribution of normalized 5hmC read counts across the gene bodies in PT and MT. **C.** Average numbers of hMRs located in different genomic regions. **D.** Heatmap clustering analysis showing the relative DNA 5hmC level of hMRs located in exons in PT and MT. Score, normalized enrichment score of 5hmC. The genes with consistent changes of 5hmC in both comparison groups are highlighted in blue boxes. **E.** Average numbers of DhMRs identified in different genomic regions. Loss indicates down-regulated 5hmC in MT; Gain indicates up-regulated 5hmC in MT. **F.** Venn diagram showing the number of DhMGs (FDR < 0.05, Log_10_ likelihood ratio ≥ 3) with 5hmC-gain in MT tissues as identified by pairwise comparison (Group 1: PT1 *vs*. MT1; Group 2: PT2 *vs*. MT2). **G**. KEGG pathway analysis of the top 3300 genes among the 6973 DhMGs shown in (F). MLN, metastatic lymph node; TSS, transcription starting site; TES, transcription ending site; hMR, 5hmC-enriched region; DhMR, differentially hydroxymethylated region; DhMG, differentially hydroxymethylated gene.

The increase of TET1/2 expression may affect the 5hmC modification levels at least in a certain number of genes. To localize the changes of 5hmC at the genomic level, we performed a genome-wide 5hmC profiling study of cancer cells for both the PT and MT groups using an approach that couples 5-hydroxymethylated DNA immunoprecipitation with deep sequencing (hMeDIP-seq). Considering possible variations of 5hmC-mediated epigenetic regulation among different molecular subtypes, we only selected ER^+^HER2^−^ invasive ductal breast cancer for hMeDIP-seq analysis. Moreover, to reduce the noise generated from histopathological heterogeneity, all fresh tissue sections were subjected to macrodissection prior to DNA extraction (Figure 2A). Two pairs of samples were subjected to hMeDIP-seq analysis (Table S1) to compare 5hmC changes between PT and MT. Similar to a previous report [16], we found that 5hmC was associated with gene-rich regions in all samples (Figure S2A and B). To determine the genome-wide distribution of 5hmC, we generated metagene profiles of normalized 5hmC read counts. 5hmC enrichment was observed throughout the gene bodies with a significant increase surrounding transcription starting sites (TSSs); moreover, 5hmC densities in MT were higher than those in PT throughout the gene bodies (Figure 2B). The hydroxymethylated regions (hMRs) were found to be enriched within the gene bodies and 1 kb upstream of TSSs (TSS-1 kb) in each sample (Table S1). Statistical analysis of hMR distribution across different genomic elements revealed that hMRs were mostly enriched in exons in all the samples (Figure 2C). Subsequent unsupervised hierarchical clustering analysis of 5hmC enrichment in exons showed that the majority of hydroxymethylated genes (hMGs) exhibited higher 5hmC levels in the MT group than in the PT group (Figure 2D).

To identify tumor metastasis-related 5hmC modifications, we performed pairwise comparison between PT1 and MT1 (Group 1), and PT2 and MT2 (Group 2) based on their clinical characteristics (tumor size, Ki-67 index, and age). On average, a total of 86,020 and 93,795 DhMRs (*P* < 0.05, Log_10_ likelihood ratio ≥  3) were identified in Group 1 and Group 2, respectively. Among them, 85,876 and 86,862 DhMRs exhibited higher 5hmC levels (DhMRs-gain) in MT1 and MT2, respectively; while only 144 and 6933 DhMRs exhibited lower 5hmC levels (DhMRs-loss) in MT1 and MT2, respectively (Table S2). Distribution analysis showed that most of the DhMRs were located in intragenic regions (Figure S2C). In contrast to DhMRs-loss that were evenly distributed in each genomic region, DhMRs-gain were primarily found in exons as well as in TSS-1 kb region (Figure 2E). To localize the gene-specific 5hmC changes between PT and MT, differentially hydroxymethylated genes (DhMGs) were identified for each pairwise comparison. Through analysis, 14,854 (DhMGs-gain, 14,828; DhMGs-loss, 26) and 14,302 (DhMGs-gain, 13,348; DhMGs-loss, 954) DhMGs were identified in Group 1 and Group 2 separately. Apparently, more than 90% of DhMGs exhibiting higher 5hmC levels in the MT samples (Table S2). Among 10,487 DhMGs-gain identified in both groups, 6973 genes exhibited higher 5hmC levels in exons (Figure 2F). Subsequent KEGG pathway  analysis showed that those genes exhibiting increased 5hmC were enriched in various pathways involved in cancer metastasis and progression including axon guidance, focal adhesion, Ras signaling, and actin cytoskeleton [5], [22] (Figure 2G; Table S3).

In summary, we established a genome-wide map of hydroxymethylome in ER^+^HER2^−^ primary tumor lesions with and without lymph node metastasis. Furthermore, our results suggest that the genome-wide gain of 5hmC landscape represents a new type of epigenetic alteration in primary tumors during the process of lymph node metastasis.

### Decreased DNA 5hmC levels in metastatic lymph node lesions

**Figure 3:**
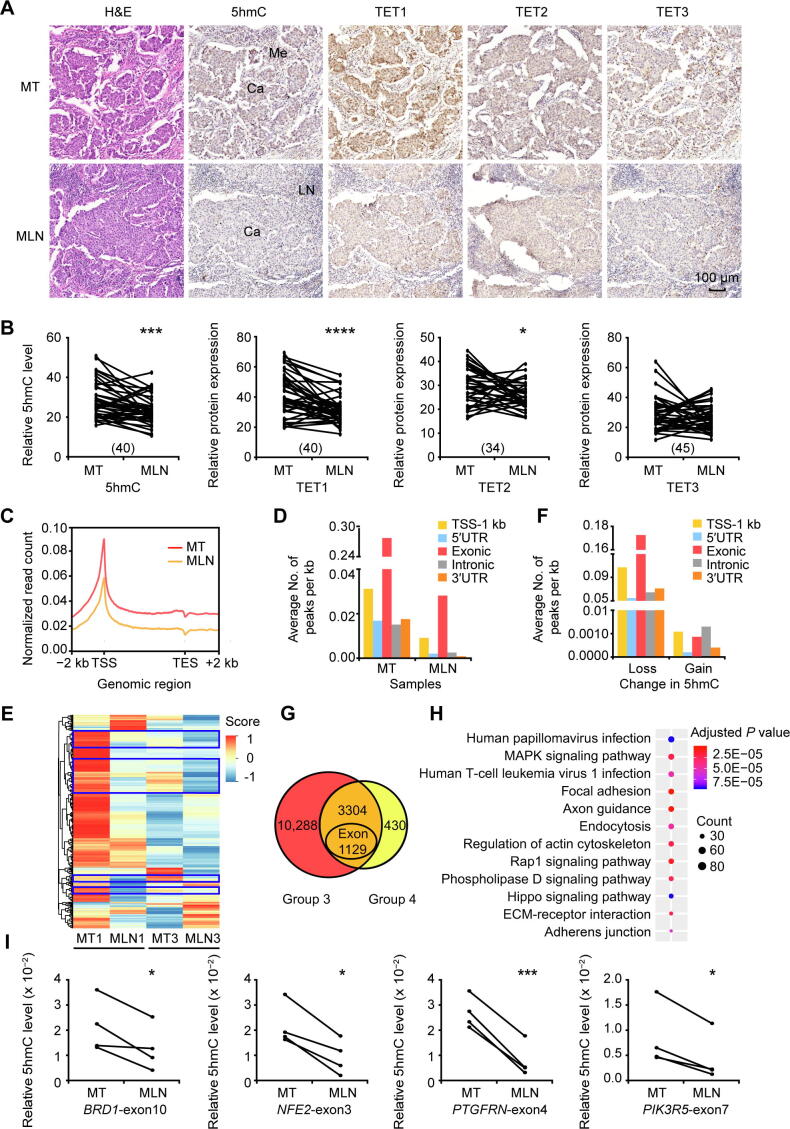
**Lower levels of DNA 5hmC and TETprotein expression inMLNlesions compared toMT lesions** **A.** Representative images of H&E staining and IHC staining to show the DNA 5hmC levels and the expression levels of TET1/2/3 proteins in MT and MLN tissues. Scale bar, 100 μm. **B.** Quantitative comparison of DNA 5hmC levels and TET1/2/3 protein expression levels between MT and MLN. The number of paired samples used for immunostaining in each group was shown in the parenthesis. **C.** Distribution of normalized 5hmC read counts across the gene bodies in MT and MLN. **D.** Average numbers of hMRs located in different genomic regions. **E.** Heatmap cluster analysis showing the DNA 5hmC level of hMRs located in exons in MT and MLN. Score, normalized 5hmC enrichment score. The genes with consistent changes of 5hmC in both comparison groups are highlighted in blue boxes. **F.** Average numbers of DhMRs identified in different genomic regions. Loss indicates down-regulated 5hmC in MLN; Gain indicates up-regulated 5hmC in MLN. **G.** Venn diagram showing the number of DhMGs (FDR < 0.05, Log_10_ likelihood ratio ≥ 3) with 5hmC-loss in MLN tissues as identified by pairwise comparison (Group 3: MT1 *vs*. MLN1; Group 4: MT3 *vs.* MLN3). **H.** KEGG pathway analysis of the 3304 DhMGs with 5hmC-loss in MLN tissues. **I.** Gene specific hMeDIP-qPCR using another 4 pairs of MT and MLN samples to validate the changes in DNA 5hmC levels in *BRD1*-exon10, *NFE2*-exon3, *PTGFRN*-exon4, and *PIK3R5*-exon7. Data are represented as mean ± SEM. *P* values are calculated by using paired Student’s *t*-test (*, *P* < 0.05; ***, *P* < 0.001; ****, *P* < 0.0001). LN represents normal lymph node tissues. MLN, metastatic lymph node.

After detaching from the primary tumor lesions, cancer cells migrate through the surrounding extracellular matrix (ECM) and form metastases inside the lymph nodes. To investigate how 5hmC changes during this process, we performed immunostaining of 5hmC and TETs in MT lesions and their matched metastatic lymph node (MLN) lesions. Intriguingly, in metastatic cancer cells of the MLN group, significant lower DNA 5hmC levels were detected, as well as lower TET1 and TET2 expression compared to those in the MT group (Figure 3A and B).

DNA hydroxymethylation is regulated by multiple factors [23], among which TET proteins are the most dominant factors responsible for that. We therefore asked whether the changes of 5hmC during breast cancer metastasis are associated with the expression of these TETs. We individually analyzed the correlation between the DNA 5hmC level and the expression of TET proteins based on their immunostaining scores in all the breast cancer samples including PT, MT, and MLN (Figure S3A–C). It was found that 5hmC levels were positively correlated with the expression levels of TET proteins, especially TET1 (*r* = 0.5719, *P* < 0.0001) (Figure S3A), suggesting that TET1 may play a dominant role in regulating the 5hmC levels during breast cancer metastasis.

To obtain a complete view of the genome-wide 5hmC changes, two MT samples and their matched MLN samples were chosen for hMeDIP-seq analysis. As shown in Figure 2A, stringent sample screening was performed to include relative pure cancer cells only for 5hmC analysis. Similar to those in PT and MT, 5hmC in the MLN samples was also distributed throughout the gene bodies with significant enrichment at TSSs (Figure 3C). Moreover, in comparison to MT, 5hmC levels were significantly decreased in MLN as judged by the normalized read counts throughout the gene bodies and TSS-1 kb. In contrast to the 59,013 and 6002 hMRs in the two MT samples, only 6845 and 1609 hMRs were identified in the two MLN samples, respectively (Table S1), most of which were located in exons (Figure 3D). Unsupervised hierarchical clustering analysis of hMRs located in exons revealed that many hMGs exhibited a significant loss of 5hmC in MLN compared to MT (Figure 3E).

To identify DhMRs between MT and MLN, pairwise comparison was performed to reduce the effect from inter-patient heterogeneity. In total, we identified 67,511 DhMRs in Group 3 (MT1 *vs*. MLN1) including 91 DhMRs-gain and 67,420 DhMRs-loss. In contrast, a comparison within Group 4 (MT3 *vs*. MLN3) revealed a total of 8397 DhMRs, which included 1213 DhMRs-gain and 7184 DhMRs-loss (Table S2). Different from DhMRs-gain, DhMRs-loss were primarily located in exons, as well as in the TSS-1 kb region (Figure 3F). The DhMRs-loss identified from the Group 3 and Group 4 were associated with 13,592 and 3734 genes, respectively, with 3304 genes (Table S2) exhibiting an apparent reduction of 5hmC levels in both groups (Figure 3G). Subsequent KEGG pathway analysis showed that the 3304 DhMGs-loss in MLN were mostly associated with human papillomavirus infection, MAPK signaling, human T-cell leukemia virus 1 infection, and focal adhesion (Figure 3H; Table S4). To examine whether the decrease in 5hmC level is a universal phenomenon in MLN, we selected several DhMGs for hMeDIP-qPCR validation by using another 4 paired samples of MT and MLN. As shown in Figure 3I and Figure S4A–D, 5hmC levels in the selected exons of *BRD1*, *NFE2*, *PTGFRN*, and *PIK3R5* were significantly reduced in MLN compared with MT.

Together, these results demonstrate that during the establishment of metastatic foci in the lymph nodes, 5hmC is lost at a genome-wide level. In addition, the different patterns of 5hmC among PT, MT, and MLN imply that 5hmC exerts different functions in distinct processes of lymph node metastasis.

### Identification of metastasis-associated 5hmC signatures as potential biomarkers for risk prediction of cancer metastasis

**Figure 4:**
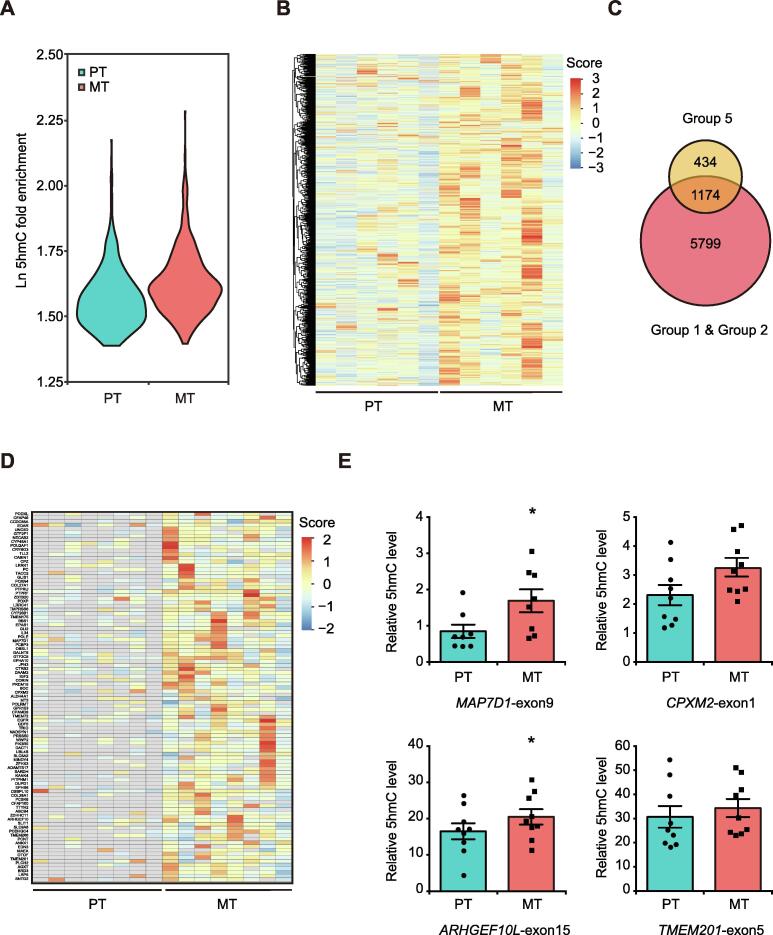
**Identification of metastasis-associated 5hmC signatures potentially used for monitoring breast cancer lymph node metastasis** **A.** Violin plots showing the relative DNA 5hmC fold enrichment of hMRs distributed in exons in additional 6 sets of PT and MT samples. **B.** Heatmap clustering analysis showing the DNA 5hmC level of all the DhMRs-gain located in exons as identified from the 6 sets of primary tumors. Score, normalized 5hmC enrichment score. **C.** Venn diagram showing the number of DhMGs exhibiting 5hmC increase in all the 8 sets of samples. (Group 5: 6 PT samples *vs*. 6 MT samples). **D.** Heatmap clustering analysis of the top 100 DhMRs showing their 5hmC levels in the 8 sets of primary tumors. Score, normalized 5hmC enrichment score. Gray indicates absence of 5hmC enrichment. **E.** Gene specific hMeDIP-qPCR to validate the changes in DNA 5hmC levels of *MAP7D1*-exon9, *ARHGEF10L*-exon15, *CPXM2*-exon1, and *TMEM201*-exon5. Data are represented as mean ± SEM. *P* values are calculated by using unpaired Student’s *t*-test (*, *P* < 0.05).

Since many genes exhibited higher 5hmC levels in MT, we then evaluated their potential to be used as biomarkers to predict cancer metastasis by performing 5hmC profiling analysis on additional 6 sets of macrodissected PT and MT samples (Table S1). In agreement with the results obtained from pairwise comparison (Figure 2), the global DNA 5hmC levels in the 6 MT samples were higher than those in the PT samples across the whole gene bodies, especially in exons (Figure 4A, Figure S5A and B). Further comparative analysis in 6 sets of PT and MT samples identified 4193 DhMRs-gain (Group 5) (FDR < 0.2), of which 2322 DhMRs-gain were located in the exons of 1608 genes. Unsupervised hierarchical clustering analysis showed that all these DhMRs-gain in exons exhibited higher 5hmC level in the 6 MT samples (Figure 4B). Similar to the DhMGs shown in Figure 2G, the 1608 genes were mostly related to MAPK signaling, focal adhesion, and regulation of cytoskeleton as revealed by KEGG pathway analysis (Figure S5C; Table S5).

By combining all the results analyzed from the 8 sets of primary tumors (Group 1, Group 2, and Group 5), a total of 1639 DhMRs corresponding to 1174 DhMGs exhibited higher 5hmC levels in MT samples than in PT samples (Figure 4C). Among them, the top 100 DhMRs with the most significant increase of 5hmC levels in MT samples were regarded as high-confidence metastasis-associated 5hmC signatures (Figure 4D). As cancer metastasis requires the activation of cytoskeleton-associated genes [22], we next selected several cytoskeleton-related genes containing metastasis-associated 5hmC signatures to confirm their 5hmC changes using hMeDIP-qPCR. Despite the inter-patient heterogeneity, *MAP7D1*-exon9 and *ARHGEF10L*-exon15 exhibited higher 5hmC levels in MT samples (Figure 4E, Figure S6A and B).

Taken together, our genome-wide profiling analysis identified a considerable number of metastasis-associated 5hmC signatures that can potentially to be used as biomarkers for predicting lymph node metastasis of breast cancer.

### MAP7D1 promotes tumor growth and metastasis in breast cancer

**Figure 5:**
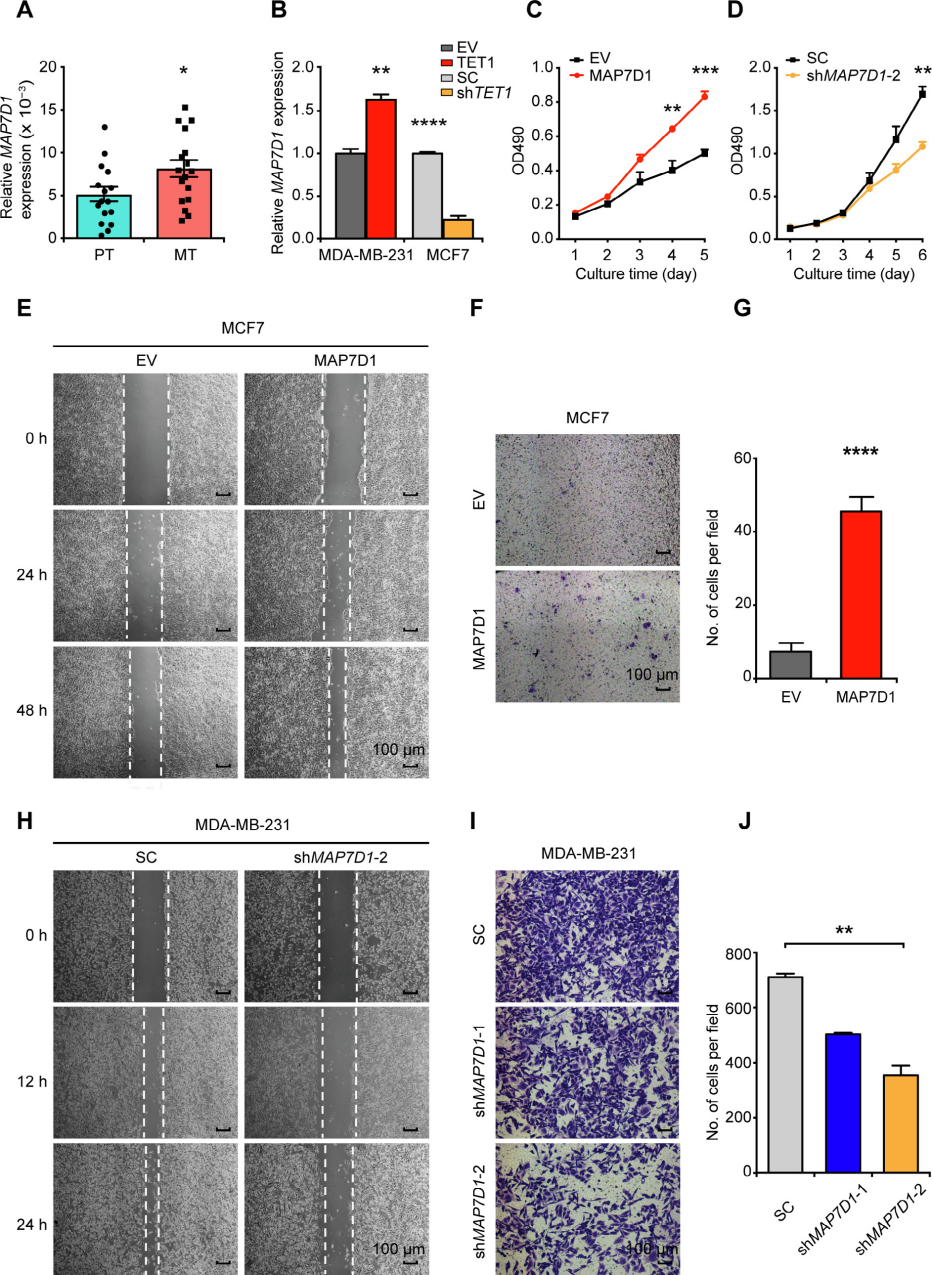
**MAP7D1 promotes breast cancer cellproliferation and invasion under the control of TET1 protein** **A.** RT-qPCR to compare the RNA expression of *MAP7D1* in 16 sets of PT and MT samples. **B.** Effect of TET1 overexpression (TET1) in MDA-MB-231 or knockdown (sh*TET1*) in MCF7 on *MAP7D1* gene expression. *18S-rRNA* was used as an internal control. **C.** and **D.** Effect of MAP7D1 overexpression in MCF7 (C) or knockdown in MDA-MB-231 (D) on cell proliferation as analyzed by MTT assays. **E.** and **H.** Representative images of wound healing assay in MCF7 (E) and MDA-MB-231 (H) to show the effect of MAP7D1 overexpression (E) or knockdown (sh*MAP7D1*-2) (H) on cell migration. Scale bar, 100 μm. **F.** and **G.** Representative photos (F) and quantitation (G) of transwell assay to show the effect of MAP7D1 overexpression on MCF7 cell migration. Scale bar, 100 μm. **I.** and **J.** Representative photos (I) and quantitation (J) of transwell assay to show the effect of MAP7D1 knockdown (sh*MAP7D1*-1 and sh*MAP7D1*-2) on MDA-MB-231 cell migration. Scale bar, 100 μm. Data are represented as mean ± SEM. *P* values are calculated by using unpaired Student’s *t*-test (*, *P* < 0.05; **, *P* < 0.01; ***, *P* < 0.001; ****, *P* < 0.0001). All the experiments were repeated three times and representative results are shown here. EV, empty vector; SC, scramble control.

Given that the levels of 5hmC in exons positively correlate with the expression levels of their respective genes [24], [25], we detected higher RNA expression of *MAP7D1* in MT samples (Figure 5A, Figure S7A–C). Moreover, among the four types of breast cancer cell lines with different metastatic abilities, the expression of *MAP7D1* was higher in two aggressive cell lines (MDA-MB-231 and MDA-MB-453), and lower in two less aggressive cell lines (MCF7 and T47D) (Figure S8A). Given the highest correlation observed between 5hmC and TET1 (Figure S3A), we asked whether *MAP7D1* is regulated by TET1. We found that overexpressed TET1 enhanced the expression of *MAP7D1*, while TET1 knockdown led to a significant decrease of *MAP7D1* expression (Figure 5B, Figure S8B and C). In contrast, overexpression of TET2 had no effect on *MAP7D1* gene expression (Figure S8D). In parallel with the increased gene expression of *MAP7D1*, a tendency of increased DNA 5hmC in *MAP7D1*-exon 9 was induced by TET1 overexpression (Figure S8E).

We further investigated the biological functions of *MAP7D1* in metastatic processes of breast cancer. We found that overexpression of MAP7D1 in MCF7 and T47D stimulated cell proliferation (Figure 5C, Figures S8F, S9A, and S9B), while MAP7D1 knockdown in MDA-MB-231 suppressed cell proliferation (Figure 5D, Figure S9C and D). In addition, overexpression of MAP7D1 promoted breast cancer cell migration (Figure 5E–G, Figure S9E and F), while MAP7D1 knockdown impaired its role on cell migration (Figure 5H–J, Figure S9G) and invasion (Figure S9H).

**Figure 6:**
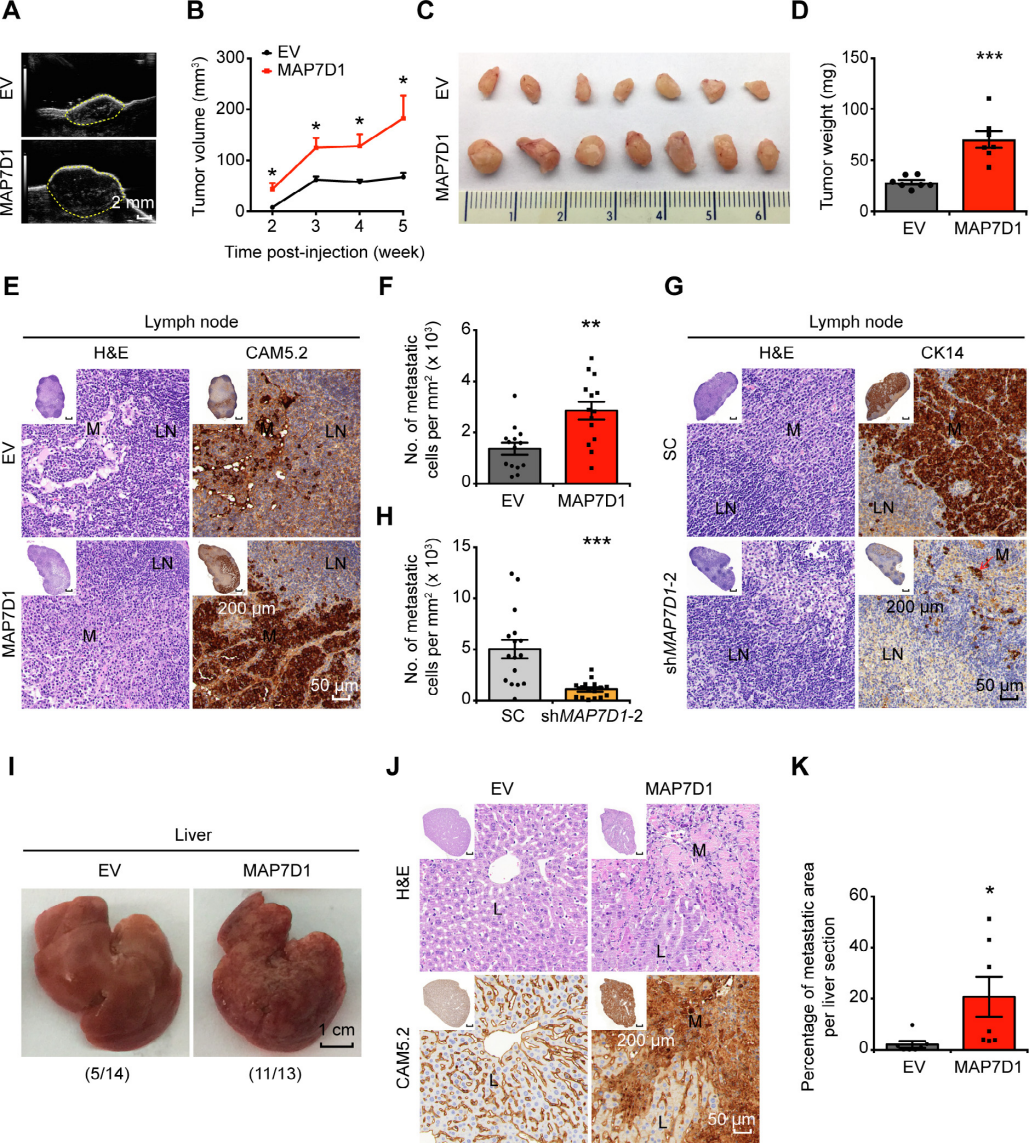
**MAP7D1 stimulates tumor growth and metastasis in the breast cancer xenograft model in nude mice** **A.** Representative ultrasound images of tumors in the control and MAP7D1 overexpression groups at the 5th week post-injection. Tumors are denoted with yellow dashed lines. **B.** Tumor growth curves in MAP7D1 overexpression group compared to the negative control. **C.** Photographs of tumors in control and MAP7D1 overexpression groups at the 5th week post-injection. **D.** Quantitative comparison of tumor weight between control and MAP7D1 overexpression groups. **E.** Representative images of H&E staining and IHC staining of CAM5.2 in MLN lesions of MCF7 xenograft mice. **F.** Quantitation of the number of metastatic cells per mm^2^ in lymph nodes based on the immunostaining results shown in (E). **G.** Representative images of H&E staining and IHC staining of CK14 in MLN lesions of MDA-MB-231 xenograft mice. **H.** Quantitation of the number of metastatic cells per mm^2^ in lymph nodes based on the immunostaining results shown in (G). **I.** Representative photographs of livers in the control and MAP7D1 overexpression groups at the 5th week post-injection. The number of mice with liver metastasis out of all the mice used in each group was shown in the parenthesis. **J.** Representative images of H&E staining and IHC staining of CAM5.2 in metastatic liver lesions of MCF7 xenograft mice. **K.** Relative ratio of metastatic area in the liver as calculated based on the immunostaining results obtained from the control and MAP7D1 overexpression groups shown in (J). Scale bar in overview images represents 200 μm, and in enlarged images represents 50 μm. In total 27 pairs of mice were utilized for the analysis, and representative data are shown here. Data are represented as mean ± SEM in each group. *P* values are calculated by using unpaired Student’s *t*-test (*, *P* < 0.05; **, *P* < 0.01; ***, *P* < 0.001). M represents metastatic cancer cells; L represents normal liver tissues.

To evaluate the *in vivo* effect of MAP7D1 on tumor growth and metastasis, we established an orthotopic breast cancer mouse model (Figure S10A) using MCF7 cells overexpressing MAP7D1 or MDA-MB-231 cells with MAP7D1 knockdown (Figures S8F and S9C). Compared to the control group, MAP7D1 overexpression significantly promoted tumor growth (Figure 6A–D), while MAP7D1 knockdown led to reduced tumor volume and weight (Figure S10B–E). Furthermore, MAP7D1 overexpression stimulated lymph node metastasis as confirmed by H&E staining and immunostaining (Figure 6E and F). However, milder lymph node metastases were observed in the knockdown group compared to their matched control (Figure 6G and H). Intriguingly, mice bearing MAP7D1-overexpressing tumors also exhibited severe liver metastasis (Figure 6I–K).

Together, we provide strong evidence here that MAP7D1, one of the representative genes containing metastasis-associated 5hmC signatures, promotes tumor growth and metastasis.

## Discussion

In this study, we investigated PT and MT lesions as well as the matched MLN lesions for their dynamic 5hmC profiles during lymph node metastasis of breast cancer. Notably, we identified a cluster of metastasis-associated 5hmC signatures as potential biomarkers for predicting lymph node metastasis. Among genes carrying these metastasis-associated 5hmC signatures, *MAP7D1* participates in breast cancer cell proliferation and metastasis.

The changes in 5hmC across various biological processes imply that 5hmC exerts diverse functions at different stages of cancer development. The higher 5hmC levels in many genes in lymph node metastasis-positive breast cancer cells indicate that reprogramming of DNA 5hmC is important for tumor development. In addition, as suitable markers to predict the risk of cancer metastasis are currently lacking, the high-confidence metastasis-associated 5hmC signatures identified in the primary tumors would allow for developing biomarkers for early detection. In comparison, the 5hmC level decreases significantly in MLN, which was also observed previously in the metastatic tissues of nasopharyngeal carcinoma, breast cancer, and colon cancer [26]. Both carcinogenesis and metastasis require well-orchestrated coordination between the intrinsic properties of cancer cells and the microenvironment. We speculate that the decrease of 5hmC in the metastatic foci may be involved in cancer cell survival in new environment and further dissemination to distant metastasis. For instance, the reduction of 5hmC in immune-related genes might impair immune response in the lymph node and thus establish metastasis.

Genome-wide profiling analysis of 5hmC in all the breast cancer samples showed that most hMRs are located within exons. Consistent with the finding that 5hmC modifications enriched in the exons positively correlate with the expression levels of their respective genes in mouse embryonic stem cells (ESCs) [24], [25]. We report here a positive correlation between the 5hmC level and the gene expression level of the cytoskeleton-related DhMG, *MAP7D1*. In addition, we demonstrated that *MAP7D1* gene expression is regulated by TET1. As a member of the MAP family [27], MAP7D1 is involved in cell adhesion, migration, cell polarity, and microtubule remodeling [28]. While its homologue MAP7D3 promotes breast cancer growth and metastasis in mice [29], the role of MAP7D1 in cancer metastasis remained unclear yet. This study provides evidence *in vitro* and *in vivo* that MAP7D1 promotes breast cancer cell proliferation and metastasis both *in vitro* and *in vivo*.

In line with dynamic changes of 5hmC, the protein expression levels of TET1 and TET2 increased in MT and then decreased in MLN. Currently, no clear consensus has been established as to whether TET proteins are oncoproteins or tumor suppressors, especially in the case of breast cancer [19], [20], [30]. The dynamic protein expression changes suggest that TETs function in a stage-dependent, dual manner during cancer development and metastasis. Of note, apart from catalyzing the oxidative DNA demethylation from 5mC to 5hmC, 5-formylcytosine (5fC), and 5-carboxylcytosine (5caC), TETs also catalyze RNA 5-hydroxymethylcytidine (5hmrC) formation [31]. Furthermore, TETs also act as transcription co-activator in regulating gene expression [32]. The versatile functions of TETs may explain why changes in protein expression of TET1 and TET2 are more significant than changes in 5hmC in all the tumor specimens. In agreement to that, overexpression of TET1 in MDA-MB-231 cells resulted in a significantly higher expression of *MAP7D1*, but only a tendency of increase in 5hmC. We therefore assume that regulation of *MAP7D1* expression by TET1 might be mediated by multiple pathways beyond DNA 5hmC.

It is interesting to note that 5hmC loss is associated with metastasis of hepatocellular carcinoma (HCC) [33]. This discrepancy with our findings is likely due to the consequence of tissue-specific regulation of 5hmC, as we found that the distribution patterns of 5hmC appear to be tissue-specific as well. Our analysis of hMR distributions revealed a significant enrichment of 5hmC surrounding TSSs, which has been reported previously in ESCs [34], [35], [36], placenta [37], and MRC5 cell line [38], but not in brain [39], colon [40], and other types of tumors [16], [17]. The context-dependent role of 5hmC indicates that its regulatory network and associated biological functions are complex and require further investigation.

In conclusion, this study depicts the dynamic 5hmC profiles during lymph node metastasis in breast cancer. Identification of metastasis-associated 5hmC signatures allows their potential use as biomarkers to predict the risk of lymph node metastasis. Furthermore, the role of MAP7D1 in breast cancer progression implies a link between 5hmC-mediated epigenetic regulation and lymph node metastasis, thus offering new options to develop diagnostic and therapeutic targets for metastatic breast cancer.

## Materials and methods

### Human breast cancer specimen

Samples of invasive ductal breast cancer and matched MLN samples were obtained from the patients who received surgery in the First Affiliated Hospital of China Medical University and then were diagnosed as invasive ductal breast cancer. Patients who received any preoperative therapy and existed distant metastasis were excluded.

### Histological and IHC staining analyses

For histological analysis, 4-μm paraffin-embedded sections or 10-μm fresh frozen tissue sections were subjected for H&E staining. For IHC staining, 4-μm paraffin-embedded sections of invasive ductal breast cancer and matched MLN samples were employed. After de-paraffinization and antigen retrieval, tissue sections were incubated with primary antibodies at 4 °C overnight and secondary antibodies at 37 °C for 2 h. After that, all the slides were counterstained with hematoxylin. The antibodies used in this study included: anti-5hmC (1:2000; catalog No. 39769, Active motif, Carlsbad, CA), anti-TET1 (1:150; catalog No. HPA019032, Sigma, St. Louis, MO), anti-TET2 (1:500; catalog No. GTX124205, Genetex, Irvine, CA), anti-TET3 (1:150; catalog No. NBP2-20602, Novus Biologicals, New York, NY), and ImmPRESSTM horse anti-rabbit IgG (Catalog No. MP-7401, Vector, Burlingame, CA).

All the images were acquired using the TissueFAXS cell analysis system (TissueGnostics, Vienna, Austria). In each image, we randomly selected at least three regions (> 1 mm^2^) that mainly contained cancer cells rather than mesenchymal cells for quantitative analysis. According to the size and ratio of length/width of each cell, the nuclei of cancer cells were identified and marked. DAB staining intensity of each cancer cell was measured by using HistoQuest software. The value in each region was calculated as the average staining intensity in all the selected cancer cells. The mean value of all selected regions was calculated and recorded as the relative level of DNA 5hmC or TET protein expression in each sample.

### Manual macrodissection of tumor specimen

For all the samples used for hMeDIP-seq, hMeDIP-qPCR, and RT-qPCR, manual macrodissection was carried out in order to get rid of mesenchymal cells or infiltrated inflammatory cells in the primary tumor, as well as lymphocytes in MLN as previously described [41]. H&E staining analysis was performed first using one 10-μm slide to identify and mark the targeted area containing tumor cells. Subsequently, its adjacent 50-μm slides were used for macrodissection by using the H&E stained, 10-μm slide as a reference.

### DNA extraction and hMeDIP-seq

For each sample, 1.5 μg of intact genomic DNA mixed with 5hmC spike-in DNA control (1:20000; catalog No. D5405-3, ZYMO Research, Irvine, CA) was used for hMeDIP. Briefly, the genomic DNA was first fragmented to a size of 100–250 bp using Covaris S220 sonicator [42] and then ligated with Illumina barcode adapter. Due to the very low binding affinity of IgG to DNA, the very little amount of DNA immunoprecipitated by IgG may cause undesirable bias among samples. Therefore, in this study we used input DNA as a control to determine the enrichment ratio of 5hmC-modified DNA. The rest was subjected to immunoprecipitation reaction with 5hmC antibody and protein-A Sepharose beads (Catalog No. P9424, Sigma) for 2 h. The concentrated 5hmC-containing DNA fragments were purified using QIAGEN Mini Elute PCR Purification Kit (Catalog No. 28004, QIAGEN, Hilden, Germany). Then, all the purified DNA fragments and input DNA were subjected to amplification, size selection (275–475 bp), and purification (Catalog No. 28704, QIAGEN) sequentially. After quality control by using Agilent Bioanalyzer 2100, all the samples (8 PT samples, 8 MT samples, and 2 pairs of MT and MLN samples) were subjected to next-generation sequencing on Illumina Hiseq X Ten system.

### Reads mapping

First, raw reads were processed with Trimmomatic (Version 0.33) to remove sequencing adaptors and low-quality bases by using default parameters [43]. The clean reads were mapped to hg19 genome by bowtie2 (Version 2.3.2) with default parameters [44]. Then SAMTools was used to remove duplicated and unpaired reads.

### Peak calling and annotation

Whole genome hMR scanning was conducted by using MACS2 (Version 2.1.1) [45]. The hMRs that fulfilled the cutoff of fold change > 4 and FDR < 0.05 were defined as significantly 5hmC-enriched regions. DhMRs were analyzed using MACS2 with default parameters (FDR < 0.05, Log_10_ likelihood ratio ≥ 3) in discovery phase, and using DiffBind (Version 3.8) package in R with FDR < 0.2 in validation phase [46]. To identify hMGs and DhMGs, hMRs and DhMRs were annotated with Annovar (Version 2016.2.1) for their locations within gene bodies and TSS-1 kb regions [47]. If both DhMRs-gain and DhMRs-loss were present in one gene, we defined the gene according to the percentage of DhMRs-gain and DhMRs-loss, which occupied more than 2/3 of the gene body in one gene. At last, the counts of hMRs and DhMRs located in TSS-1 kb, 5′UTR, exon, intron, 3′UTR, and intergenic regions were normalized by the length of these elements.

### KEGG pathway enrichment analysis

KEGG pathway enrichment analysis of the selected DhMGs was carried out using the clusterProfiler (Version 3.8.1) package in R [48]. The cut-off value of FDR for the significant enrichment pathway was 0.05.

### Visualization

For each sample, the hMR peak profiles in bed format for individual genes were visualized by using Integrative Genomics Viewer (IGV) [49]. The average read counts per million distribution of the gene was displayed from 2 kb upstream of TSS to 2 kb downstream of transcription ending site (TES) using deeptools [50]. KEGG pathway enrichment and violin plot of 5hmC enrichment were plotted by R package ggplot2 (Version 3.1.0) [51]. Clustering and heatmap plotting of hMRs or DhMRs were conducted by pheatmap package (Version 1.0.10) in R.

### hMeDIP-qPCR

For gene-specific hMeDIP-qPCR, 6 μg of genomic DNA was fragmented to a size of 500–800 bp [52] using Covaris S220 sonicator and then immunoprecipitated as described above. To determine the enrichment ratio of 5hmC-modified DNA fragments, an equal ratio of input and hMeDIP products were used as templates for RT-qPCR with THUNDERBIRD™ SYBR® qPCR Mix (Catalog No. QPS-201, TOYOBO, Osaka, Japan). Relative enrichment of 5hmC was calculated as (1/2)^dCt^ (dCt = Ct_IP_ − Ct_input_). In total, 8–10 sets of PT and MT samples as well as 4 sets of MT and MLN samples were utilized for hMeDIP-qPCR. All the hMeDIP experiments were performed at least in two technical repeats and qPCR of each sample was performed in three technical repeats.

### RNA extraction and RT-qPCR

Total RNA was extracted from macrodissected samples or cultured cells by using TriReagent (Catalog No. T92424, Sigma). 1 μg of RNA was used for cDNA synthesis with ReverTra Ace® qPCR RT Master Mix (Catalog No. FSQ-301, TOYOBO) and RT-qPCR was carried out to determine the gene expression level. All the primers used in the experiments are listed in Table S6. In total, 8–16 sets of PT and MT samples were utilized for mRNA detection. All the experiments were performed in three technical replicates.

### Plasmid construction and lentivirus packaging

The FH-TET1-pEF and pcDNA3-hTET2 plasmids were kindly provided by Prof. Huang Bo (Institute of Basic Medical Sciences, Chinese Academy of Medical Sciences & Peking Union Medical College, China). The pLV-hMAP7D1 plasmid and its empty vector pLV-EV were purchased from Vector Builder Company (Beijing, China). The sequence of *hTET1* shRNA was 5′-aacccGCAGCTAATGAAGGTCCAGAAttcaagagaTTCTGGACCTTCATTAGCTGCtttttc-3′ [53]. Two sets of shRNAs targeting *MAP7D1* were utilized in this study: sh*MAP7D1*-1, 5′-aacccGCTGGAGGAGCAACGTCTTAAttcaagagaTTAAGACGTTGCTCCTCCAGCtttttc-3′; sh*MAP7D1*-2, 5′-aacccGACTCGGAAGTCAGAAGTTTCttcaagagaGAAACTTCTGACTTCCGAGTCtttttc-3′. shRNAs of human *TET1* and *MAP7D1* were cloned into pLL3.7 vector between *Hpa*I and *Xho*I restriction enzyme sites.

The lentiviral knockdown system (including pLL3.7, pRSV-rev, pMDLg/pRRE, and pCMV-VSV-G) and the lentiviral overexpression system (including pLV, pH1, and pH2) were utilized for lentivirus package in 293 T cells as described previously [54].

### Cell culture, plasmid transfection**,** and lentivirus infection

Breast cancer cell lines were purchased and authenticated from National Infrastructure of Cell Line Resource (Beijing, China). MCF7 and 293T were cultured in DMEM; MDA-MB-231 and MDA-MB-453 were cultured in L15; T47D were cultured in RPIM 1640. All the culture media were supplemented with 10% fetal bovine serum (FBS) and 1% penicillin/streptomycin. Transfection of 293T and MCF7 cells was accomplished using DNA transfect reagent (Catalog No. TF201201, Neofect, Beijing, China) according to the manufacturer’s instructions. Electroporation (Catalog No. NEPA21, NEPAGENE, Chiba, Japan) and lentivirus infection were performed with MDA-MB-231 and T47D cells.

### Cell proliferation assay

2.5 × 10^3^ MDA-MB-231 cells or 4 × 10^3^ MCF7/T47D cells per well were seeded into 96-well culture plates. Cell proliferation was determined by using MTT assay (Catalog No. M1020-500, Solarbio, Beijing, China) in accordance with the manufacturer’s instructions.

### Wound healing assay

Cells were seeded into 6-well plates at a density that cells could reach confluence 48–72 h post transfection. Complete medium was then replaced with 1% FBS supplemented medium to prevent further proliferation. A scrape wound was made in each well, and the cells were photographed at 0 h, 12 h, and 24 h for MDA-MB-231 and 0 h, 24 h, and 48 h for MCF7 and T47D. Images were acquired under a Nikon ECLIPSE Ti microscope (Santa Clara, CA).

### Transwell assay

For cell migration assay, MCF7, T47D or MDA-MB-231 cells were seeded into the upper chambers containing serum-free medium at a density of 5 × 10^5^ cells (MCF7/T47D) or 2.5 × 10^4^ (MDA-MB-231) per well. Complete medium supplemented with 30% FBS (MCF7/T47D) or 10% FBS (MDA-MB-231) was added to the lower chambers. After being cultured for 24 h (MDA-MB-231), 72 h (MCF7) or 48 h (T47D), cells on the lower chambers of transwell membranes were fixed with 70% ethanol and stained with 1% crystal violet. Images of the stained cells were acquired under Nikon ECLIPSE Ti microscope. Crystal violet-stained cells were counted in six random fields at ×40 (MCF7/T47D) and ×100 (MDA-MB-231).

Cell invasion assay was performed by using the transwell chambers coated with extracellular matrix (Catalog No. 356234, BD, San Jose, CA). 5 × 10^5^ MDA-MB-231 cells were seeded into the upper chamber and incubated with 30% FBS supplemented medium for 48 h. Then the cells on the lower chamber were stained and counted as described above.

### Xenograft tumor assays in nude mice

4–5 week-old female BALB/C nude mice were purchased from Vital River company (Beijing, China) and housed in a specific pathogen-free condition under a 12 h light/12 h dark cycle at 23 °C with food and water. Orthotopic mammary fat pad xenografts were established by injecting 2 × 10^6^ MDA-MB-231 cells or 1 × 10^7^ MCF7 cells suspended in the mixture of PBS and Matrigel (Catalog No. 354230, BD) (PBS: Matrigel = 1:1) into the fourth fat pad of nude mice. For mice receiving MCF7 cells, subcutaneous injection of 100 μl of estrogen was performed once a week. Tumor volume was measured every week and calculated as length × (width)^2^/2 based on ultrasound imaging analysis (VisualSonics vevo 2100 Imaging System, FUJIFILM, Toronto, Canada). After injection for 4–7 weeks (MDA-MB-231) or 5–6 weeks (MCF7), mice were sacrificed and examined for tumor growth and metastasis to lymph node, liver, and lung. Paraffin-embedded blocks were prepared for all the tumors and the aforementioned organs for H&E analysis. In addition, to precisely evaluate the metastatic status, the metastatic nodules in lymph node and liver were also assessed by IHC staining with luminal cell marker CAM5.2 (Catalog No. ZM-0316, ZSGB-BIO, Beijing, China) for MCF7 and myoepithelial cell marker CK14 (Catalog No. RTU-LL002, Leica, Buffalo Grove, IL) for MDA-MB-231. The extent of metastasis was compared based on the ratio of metastatic area in the liver, and the number of metastatic cells per mm^2^ in lymph node, as analyzed by using HistoQuest software. In total, 27 pairs of mice were used for the experiment.

### Statistical analysis

Unpaired Student’s *t*-test analysis was applied for statistical analyses in Figures 1B, 4E, 5A–D, 5G, 5J, 6B, 6D, 6F, 6H, and 6K and Figures S6B, S7A–C, S8B–F, S9A–D, S9F, S9H, S10C, and S10E. Paired Student’s *t*-test was used to measure the variables between MT and MLN in Figure 3B and I. The correlations between DNA 5hmC level and the protein expression levels of TET1, TET2, and TET3 in Figure S3A–C were assessed using Pearson test. All the data are represented as mean ± SEM. *P* < 0.05 is considered statistically significant.

## Ethical statement

All the human tissue samples were obtained under a protocol (AF-SOP-07-1.1-01) approved by the Medical Scientific Research Ethics Committee of the First Affiliated Hospital of China Medical University. All subjects provided written informed consent according to the Institutional Guidelines. This study is compliant with all relevant ethical regulations regarding research involving human resources. All animal experiments and euthanasia were approved and performed in accordance with the guidelines of Animal Care and Use Committee of IBMS/PUMC. The Institutional Review Board (IRB) approval number is ACUC-A02-2018-007.

## Data availability

The datasets have been deposited in the Sequenced Read Archive (SRA: SRS3995487–SRS3995493 and SRS4479368–SRS4479379), and are publicly accessible at https://www.ncbi.nlm.nih.gov/sra. The datasets have also been deposited in the Genome Sequence Archive [55] at the National Genomics Data Center, Beijing Institute of Genomics, Chinese Academy of Sciences / China National Center for Bioinformation (GSA: CRA001593), and are publicly accessible at http://bigd.big.ac.cn/gsa.

## CRediT author statement

**Shuang-Ling Wu:** Methodology, Validation, Investigation, Writing - original draft. **Xiaoyi Zhang:** Formal analysis, Data curation, Writing - original draft. **Mengqi Chang:** Methodology. **Changcai Huang:** Data curation. **Jun Qian:** Data curation. **Qing Li:** Resources. **Fang Yuan:** Methodology. **Lihong Sun:** Methodology. **Xinmiao Yu:** Resources. **Xinmiao Cui:** Resources. **Jiayi Jiang:** Resources. **Mengyao Cui:** Resources. **Ye Liu:** Resources. **Huan-Wen Wu:** . **Zhi-Yong Liang:** . **Xiaoyue Wang:** Formal analysis. **Yamei Niu:** Conceptualization, Supervision, Funding acquisition, Writing - original draft. **Wei-Min Tong:** Conceptualization, Project administration, Writing - review & editing. **Feng Jin:** Supervision, Project administration, Funding acquisition. All authors read and approved the final manuscript.

## Competing interests

Institute of Basic Medical Sciences Chinese Academy of Medical Sciences has filed patent application based on this work, and the patent number is 201910187035.3.

## Supplementary material

Supplementary Figure S1 **Representative images showing quantitative analysis of immunostaining intensity in breast cancer cells (Related to Figure 1)**. **A**. Representative images showing the nuclear staining of 5hmC in breast cancer tissues (red rectangle) and normal tissues (black rectangle) in the same paraffin section. The area of nuclei was denoted in red circle with red arrow. Ca, invasive ductal breast cancer cells; N, normal epithelial cells; Me, mesenchymal cells. Scale bar in overview images represents 200 μm, and in enlarged images represents 25 μm. **B**. Representative images showing the way to calculate score values for immune-stained samples. By using HistoQuest software, the nuclei of cancer cells (denoted in red circle) were recognized and included for downstream analysis. The nuclei of non-tumor cells are denoted in green. Scale bar represents 200 μm. **C**. Examples of cells with different staining intensities are shown in three boxes. The cells selected for analysis were denoted in red cycles, including strong staining (red arrow, 1#), medium staining (black arrow, 2#), and weak staining (blue arrow, 3#) (left panel). Each spot in the right panel represents one individual cell selected for analysis. Y-axis represents the “staining intensity” of each cell. The score value of each sample was calculated by the average staining intensities of all the selected cells. Scale bar represents 10 μm.

Supplementary Figure S2 **Genome-wide distribution of 5hmC in gene bodies and intergenic regions (Related to Figure 2)**. **A**. IGV plots showing 5hmC distribution in the region of chr16: 46,988,262-60,315,587. Gray reads originate from input libraries; Blue and red reads originate from hMeDIP libraries of PT and MT, respectively. Y-axis represents normalized number of read counts. **B**. Statistical analysis of hMRs located in TSS-1 kb, gene bodies, and intergenic regions in PT and MT. **C**. Statistical analysis of DhMRs between PT and MT located in TSS-1 kb, gene bodies and intergenic regions. Loss, down-regulated 5hmC in MT; Gain, up-regulated 5hmC in MT.

Supplementary Figure S3 **Correlation analysis between global DNA 5hmC levels and TET1/ TET2/ TET3 protein expression levels (Related to Figure 3)**. The Spearman correlation between global DNA 5hmC levels and TET1 (**A**), TET2 (**B**), and TET3 (**C**) as calculated from immunohistochemical staining scores as shown in Figure 1B and 3B. The number of samples used for analysis was shown in each figure.

Supplementary Figure S4 **IGV plots showing the 5hmC profiles of the genes validated for 5hmC decrease from MT to MLN (Related to Figure 3)**. IGV plots showing 5hmC distribution in *BRD1*-exon10 (**A**), *NFE2*-exon3 (**B**), *PTGFRN*-exon4 (**C**), and *PIK3R5*-exon7 (**D**) in MT and MLN, respectively. Gray reads originated from input libraries; red and purple reads originated from hMeDIP libraries of MT and MLN, respectively. Y-axis represented normalized numbers of read counts. The enlarged view of the 5hmC profile in the selected exons (denoted with red arrows) is shown in the right panel of each Figure.

Supplementary Figure S5 **hMeDIP-seq analysis of additional 6 sets of PT and MT samples (Related to Figure 4)**. **A**. Metagene profile analysis showing the distribution of hMRs across the gene bodies. **B**. Violin plot showing the relative DNA 5hmC fold enrichment. **C**. KEGG analysis of the 1608 DhMGs exhibiting higher 5hmC levels in the 6 MT samples.

Supplementary Figure S6 **IGV plots showing the 5hmC profiles of the genes validated for 5hmC increase** in **MT****compared to PT****(Related to Figure 4)**. **A**. IGV plots showing 5hmC distribution in *MAP7D1*-exon9, *ARHGEF10L*-exon15, *CPXM2*-exon1, and *TMEM201*-exon5. Gray reads originated from input libraries; blue and red reads originated from hMeDIP libraries of PT and MT, respectively. *Y-axis* represented normalized numbers of read counts. The enlarged view of the 5hmC profile in the selected exons (denoted with red arrows) is shown in the right panel. **B**. The relative 5hmC fold enrichment of the four genes in selected exons as acquired from 8 sets of hMeDIP-seq data. *, *P* < 0.05.

Supplementary Figure S7 **RT-qPCR analysis to determine the RNA expression levels of the three genes validated for 5hmC increase from PT to MT (Related to Figure 4)**. RT-qPCR analysis to determine the RNA expression of *ARHGEF10L* (**A**), *CPXM2* (**B**), and *TMEM201* (**C**) in the 8 sets of PT and MT samples. *18s-rRNA* was used as an internal control. Data are represented as mean ± SEM, *P* values were calculated by using unpaired student’s *t*-test. *, *P* < 0.05.

Supplementary Figure S8 **Validation of overexpression and knockdown efficiency of TET1 and MAP7D1 in breast cancer cell lines (Related to Figure 5). A**. Relative RNA expression levels of MAP7D1 in four types of breast cancer cell lines. 231, MDA-MB-231; 453, MDA-MB-453. *β*-actin was used as an internal control. **B**. RT-qPCR analysis to confirm the overexpression efficiency of TET1 in MDA-MB-231 cell line. **C**. RT-qPCR analysis to confirm the knockdown efficiency of TET1 in MCF7 cell line. **D**. RT-qPCR analysis to determine the effect of TET2 overexpression on *MAP7D1* gene expression in MDA-MB-231 cell line. **E**. hMeDIP-qPCR analysis to determine the level of 5hmC in *MAP7D1*-exon9 after TET1 overexpression in MDA-MB-231 cell line. **F**. RT-qPCR analysis to validate the efficiency of MAP7D1 overexpression in MCF7. EV, empty vector; SC, scramble control. *18s-rRNA* was used as an internal control. Data are represented as mean ± SEM, *P* values were calculated by using unpaired student’s *t*-test, ***, *P* < 0.001; ****, *P* < 0.0001. All the experiments were repeated three times and representative results are shown here.

Supplementary Figure S9 **Effect of MAP7D1 on breast cancer cell proliferation and migration (Related to Figure 5)**. **A**. RT-qPCR analysis to validate the efficiency of *MAP7D1* overexpression in T47D. **B**. Effect of *MAP7D1* overexpression in T47D on cell proliferation as analyzed by MTT assays. **C**. RT-qPCR analysis to validate the efficiency of *MAP7D1* knockdown in MDA-MB-231. Sh*MAP7D1*-1 and sh*MAP7D1*-2 represent two kinds of plasmids containing different shRNA sequence. **D**. Effect of *MAP7D1* knockdown in MDA-MB-231 on cell proliferation as analyzed by MTT assays. Representative images of wound healing assay in T47D and MDA-MB-231 cells to show the effect of *MAP7D1* overexpression (**E**) or knockdown (sh*MAP7D1*-1) (**G**) on cell migration. **F**. Representative images and quantitative analysis of transwell assay to show the effect of *MAP7D1* overexpression on T47D cell migration. **H**. Representative images and quantitative analysis of transwell assay to show the effect of *MAP7D1* knockdown on MDA-MB-231 cell invasion. EV, empty vector; SC, scramble control. Scale bar represents 100 μm. *18s-rRNA* was used as an internal control. Data are represented as mean ± SEM, *, *P* < 0.05; **, *P* < 0.01; ****, *P* < 0.0001. All the experiments were repeated three times and representative results are shown here.

Supplementary Figure S10 **Effect of MAP7D1 on tumor metastasis as observed in breast cancer xenograft model in nude mice (Related to Figure 6)**. **A**. Schematic representation of orthotropic xenograft assay. MCF7 and MDA-MB-231 cell lines with stable MAP7D1 overexpression or knockdown were established by using clone selection and lentivirus system, respectively. Then the cells were injected into the inguinal mammary fat pad. Tumor growth was monitored once every week via ultrasound imaging analysis. In order to evaluate the progression of metastasis, primary tumors (red arrows), lymph nodes (blue arrows), livers as well as lungs were included for gross observation, H&E staining and immunohistochemical staining analysis. **B**. Representative ultrasound images of tumors in the SC and sh*MAP7D1*-2 groups at the 3^rd^ week post-injection. Tumors are denoted with yellow dashed lines. **C**. Tumor growth curves in the sh*MAP7D1*-2 group compared to that in the SC group. **D**. Photographs of tumors in SC and sh*MAP7D1*-2 groups at the 4^th^ week post-injection. **E**. Quantitative comparison of tumor weight between SC and sh*MAP7D1*-2 groups at the 7^th^ week post-injection. Data are represented as mean ± SEM in each group. *P* value was calculated by using unpaired student’s *t*-test. *, *P* < 0.05.
